# Erasing the Epigenetic Memory and Beginning to Switch—The Onset of Antigenic Switching of *var* Genes in *Plasmodium falciparum*

**DOI:** 10.1371/journal.pone.0034168

**Published:** 2012-03-26

**Authors:** Yair Fastman, Robert Noble, Mario Recker, Ron Dzikowski

**Affiliations:** 1 Department of Microbiology & Molecular Genetics, The Kuvin Center for the Study of Infectious and Tropical Diseases, IMRIC, The Hebrew University-Hadassah Medical School, Jerusalem, Israel; 2 Department of Zoology, University of Oxford, Oxford, United Kingdom; Weill Cornell Medical College, United States of America

## Abstract

Antigenic variation in *Plasmodium falciparum* is regulated by transcriptional switches among members of the *var* gene family, each expressed in a mutually exclusive manner and encoding a different variant of the surface antigens collectively named PfEMP1. Antigenic switching starts when the first merozoites egress from the liver and begin their asexual proliferation within red blood cells. By erasing the epigenetic memory we created parasites with no *var* background, similar to merozoites that egress from the liver where no *var* gene is expressed. Creating a null-*var* background enabled us to investigate the onset of antigenic switches at the early phase of infection. At the onset of switching, *var* transcription pattern is heterogeneous with numerous genes transcribed at low levels including upsA *vars*, a subtype that was implicated in severe malaria, which are rarely activated in growing cultures. Analysis of subsequent *in vitro* switches shows that the probability of a gene to turn on or off is not associated with its chromosomal position or promoter type *per se* but on intrinsic properties of each gene. We concluded that *var* switching is determined by gene specific associated switch rates rather than general promoter type or locus associated switch rates. In addition, we show that fine tuned reduction in *var* transcription increases their switch rate, indicating that transcriptional perturbation can alter antigenic switching.

## Introduction

*Plasmodium falciparum* causes the deadliest form of human malaria and its virulence is attributed in part to the ability of the parasites to undergo antigenic variation of immunodominant cell surface proteins to avoid immune recognition [1], [2], [3].

The major antigenic ligands responsible for cytoadhesion are members of the highly polymorphic PfEMP1 [4] proteins expressed on the surface of infected red blood cells (iRBC). The placement of parasite-encoded proteins on the cell surface stimulates an antibody response that often clears the majority of infected cells from the circulation. However, small sub-populations of parasites switch expression to an alternative PfEMP1 variant [5] and thus avoid the antibody response and re-establish the infection. This process of antigenic variation is responsible for the persistent nature of the disease as well as the waves of parasitemia frequently observed in *P. falciparum* infections [6]. PfEMP1 are encoded by a multi-copy gene family named *var* [7] and each parasite genome has a repertoire of ∼60 *var* genes [8]. Different parasite isolates typically have completely different *var* complements resulting in a virtually limitless repertoire of antigenic determinants [9]. Despite the high variability of the *var* gene family its genomic organization is well conserved across different parasite isolates. Approximately two thirds of the *var* genes are located in subtelomeric regions and the rest are found in the internal regions of the chromosomes. *Var* genes have been classified into several subgroups based on the sequence similarity of their 5′ UTRs [10], [11] with subtelomeric *var* genes having either upsA or upsB promoters, whereas centrally located *var* genes have upsC promoters; *var2csa*, the *var* gene that was implicated in pregnancy associated malaria, has a unique upsE promoter. The proportions of these groups seem to be highly conserved between different parasite isolates [12], [13].

Immune evasion of *P. falciparum* depends on its ability to express only a single *var* gene at a time in order to limit antigenic exposure [14], [15], and to undergo intrinsic transcriptional switches between different *var* genes [16]. These switches need to occur often enough to generate parasite subpopulations that escape the human immune response, yet also need to be tightly controlled in order to avoid premature expenditure of the 60 different proteins. Horrocks *et al*. [17] were the first to suggest the existence of a hierarchy in *var* gene switching and showed that different *var* genes have different intrinsic switch rates [17]. These rates were later proposed to be associated with their chromosomal location [18]. More recently, models have demonstrated that this highly structured switching pattern, based on different switch rates and activation probabilities, is crucial to prevent exhaustion of the antigenic repertoire in the early stages of infection [19].

Passage of *P. falciparum* through the liver of the mammalian host appears to reset the *var* gene expression pattern. A parasite population recovered from a patient only 11 days post infection indicated that most *var* genes were expressed at this stage [20]. Similar results were obtained when parasite populations recovered from human volunteers were put in cultures for a month prior to transcriptional analysis [21]. However, obtaining conclusive data on the entire *var* expression profile in early merozoites that emerge from the liver is challenging since experiments done in human volunteers requires treatment when low parasitemia is observed and such low parasitemia does allow recovering enough RNA for transcriptional profiling. Thus far no experimental system exists that enables studying the onset of antigenic switching in the very early merozoites that egress from the liver and enter the blood stream at the early phase of infection. This represents a fundamental deficiency in our ability to understand parasite biology since the ability to establish an initial blood stage infection is of paramount importance for disease transmission.

Previously, we have demonstrated a new procedure to silence all *var* genes in the genome by promoter titration [22]. However, transcription patterns were documented only 10 weeks after switching initiation and therefore *var* gene expression at the onset of switching remained unknown. The ability to silence all *var* genes in a reversible way and erase their epigenetic memory enables us for the first time to study *in vitro var* expression at the onset of antigenic switching and thus, mimicking the moment when parasites exit the liver and first invade RBCs. In addition, this procedure allowed us to fine tune interference with *var* transcription and provided new insights for the possible mechanisms that influence switch rates that regulate antigenic variation.

## Results

### Erasing epigenetic memory and beginning to switch: At the onset of antigenic switching var transcription pattern is heterogeneous

**Figure 1:**
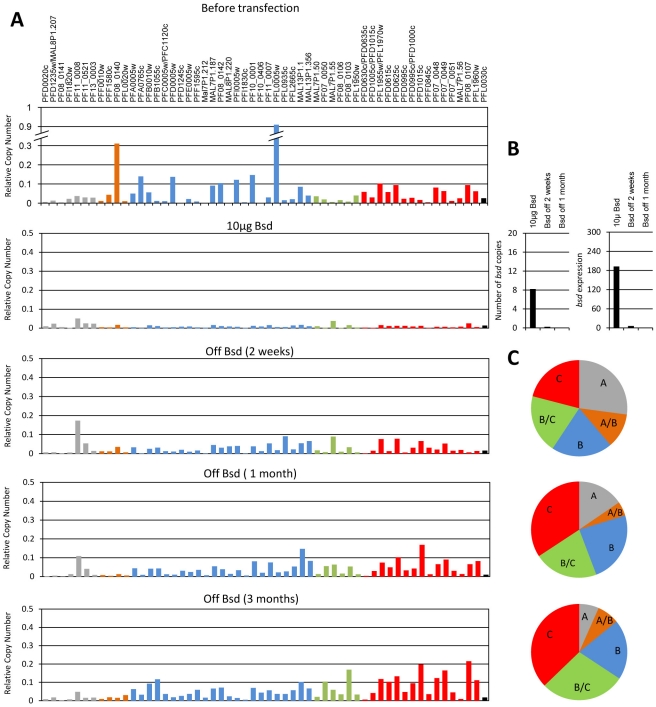
Erasing the epigenetic memory of the entire *var* gene family by promoter titration and the onset of switching of the E-1 population. (**A**), Steady state mRNA levels of each individual *var* gene are presented as relative copy number. All values are presented as relative copy number to the housekeeping genes arginyl-tRNA synthetase (PFL0900c), fructose biphosphate aldolase (PF14_0425), and actin (PFL2215). Each *var* gene is colored by its promoter type: Grey, upsA; Orange, upsA/B; Blue, upsB; Green, upsB/C; Red, upsC; Black, upsE. Transcription profiles are presented before transfection (upper panel), under titration using selection on 10 µg/ml blasticidin (second panel), two weeks, one month and three months (third to lower panel respectively) after drug removal and switching initiation. (**B**), pVBH copy numbers (left panel) and *bsd* transcription levels (right panel) of the E-1 populations under selection on 10 µg/ml blasticidin, as well as at two weeks and one month after drug removal. The number of pVBH copies was measured by qPCR on gDNA and transcription levels were measured by RT-qPCR on cDNA. (**C**), Dynamics in the relative transcription of each *var* gene subsets two weeks, one month and three months after drug removal (upper to lower pies respectively). The relative proportion of each *var* gene subsets, A, A/B, B (telomeric) and B/C, C (central) from the total transcripts is displayed for each population at each time points. The relative proportion of transcript was normalized to the number of genes belonging to each *var* subset.

**Figure 2:**
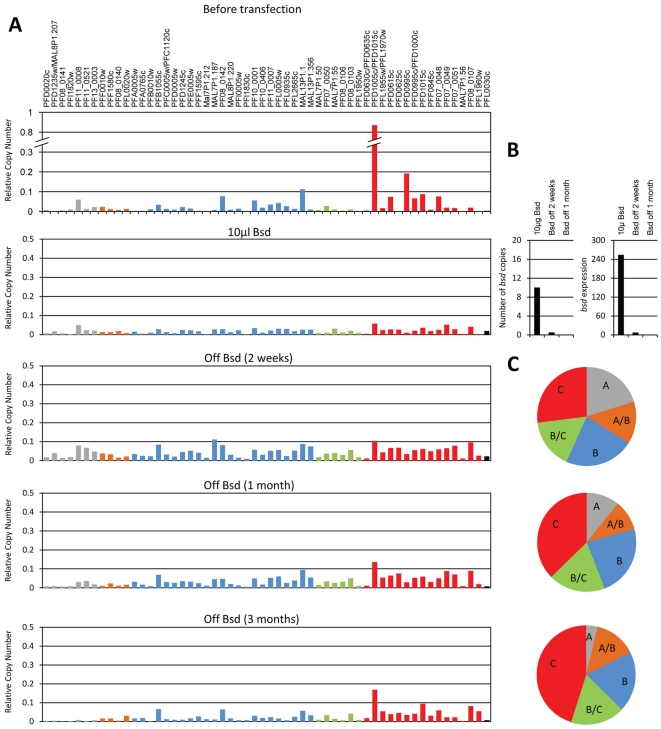
Erasing the epigenetic memory of the entire *var* gene family by promoter titration and the onset of switching of the G-6 population. (**A**), Steady state mRNA levels of each individual *var* gene are presented as relative copy number. All values are presented as relative copy number to the housekeeping genes arginyl-tRNA synthetase (PFL0900c), fructose biphosphate aldolase (PF14_0425), and actin (PFL2215). Each *var* gene is colored by its promoter type: Grey, upsA; Orange, upsA/B; Blue, upsB; Green, upsB/C; Red, upsC; Black, upsE. Transcription profiles are presented before transfection (upper panel), under titration using selection on 10 µg/ml blasticidin (second panel), two weeks, one month and three months (third to lower panel respectively) after drug removal and switching initiation. (**B**), pVBH copy numbers (left panel) and *bsd* transcription levels (right panel) of the G-6 populations under selection on 10 µg/ml blasticidin, as well as at two weeks and one month after drug removal. The number of pVBH copies was measured by qPCR on gDNA and transcription levels were measured by RT-qPCR on cDNA. (**C**), Dynamics in the relative transcription of each *var* gene subsets two weeks, one month and three months after drug removal (upper to lower pies respectively). The relative proportion of each *var* gene subsets, A, A/B, B (telomeric) and B/C, C (central) from the total transcripts is displayed for each population at each time points. The relative proportion of transcript was normalized to the number of genes belonging to each *var* subset.

In order to investigate the onset of *var* gene switching activation we used promoter titration, briefly described below, to silence and erase the epigenetic memory of different parasite populations and thus created parasite lines with a null-*var* expression background similar to the initial merozoite population at the beginning of blood stage infection. The first clone that we erased expressed a subtelomeric *var* gene with an upsB promoter (E-1, expressing predominantly PFL0005w); the second line expressed a central *var* gene with upsC promoter (G-6, expressing predominantly PFD1005c). Both clones, each expressing a different PfEMP1 have similar growth rate which is also similar to the growth rate of transgenic parasite lines expressing no PfEMP1 (unpublished data). This fact argues that dynamics in transcription patterns of each population reflects switch rates rather than fitness cost of particular PfEMP1 expression. Each of these parasite lines was transfected with the pVBH plasmid by electroporation as described [23] and selected on 10 µg/ml blasticidin for populations that carry high copy numbers of active episomal *var* promoters. The competing pVBH episomes effectively silenced the predominant *var* genes, PFL0005w in the E-1 clone (Fig. 1A, second panel) and PFD1005c in the G-6 clone (Fig. 2A, second panel), by promoter titration. Both parasite populations transcribed high levels of the *bsd* gene from ∼8–10 episomal copies of pVBH (Fig. 1B & 2B).

In order to allow *in vitro* switching to begin we removed drug pressure from the transfected populations, thus enabling the parasites to shed the pVBH episomes. qPCR of parasite populations growing for one month under no drug pressure indicated that these parasites no longer carried the pVBH plasmid and did not express *bsd* (Fig. 1B & 2B). We then followed the switching patterns of the entire *var* gene family of these parasite populations over a period of half a year (92 generations) using RT-qPCR as described [22]. RNA was extracted every two weeks for the first 3 months and then once a month until the end of the experiment. Two weeks (7 generations) after drug pressure was removed low levels of endogenous *var* transcripts could be detected, indicating that the parasites had started to actively express endogenous *var* genes. At this early time point, low transcription levels of many genes could be detected both in the E-1 and the G-6 population (Fig. 1A & 2A, third and fourth panels respectively), indicating a high activation probability of these genes from a null-*var* background. In both the E-1 and the G-6 populations transcription of upsA *var* genes was also detected in the early generations (up to one month) following switching initiation indicating that these genes can be activated from a null-*var* background at the early phase of infection. Moreover, at the onset of switching (up to one month after switching initiation) the relative proportion of transcript levels per *var* gene was similar among the different *var* subtypes, including upsA, both in the E-1 and G-6 populations (Fig. 1C & Fig. 2C).

### Switching dynamics of different var subsets are independent of chromosomal location and promoter type per se

**Figure 3:**
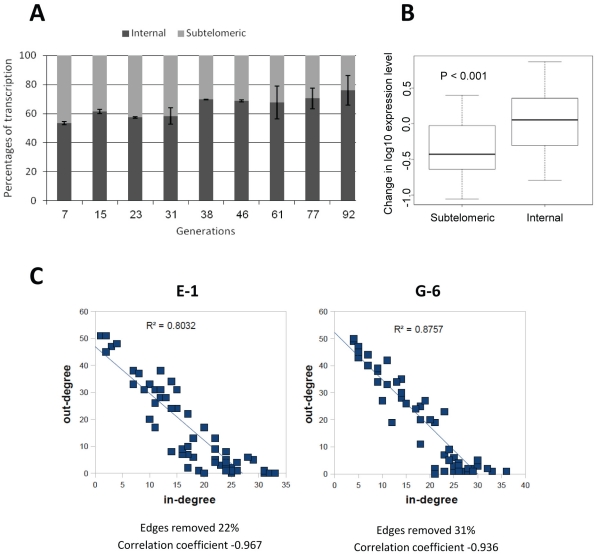
Switching dynamics of *var* genes in the following generations after erasing the epigenetic memory are biased. (**A**) Over time *var* transcription is biased toward internal *var* genes. The ratio of the mean total transcripts from internal and subtelomeric *var* genes (normalized to the number of genes in each sub-group) of the E-1 and G-6 cultures is presented for each time point. Error bars represents standard errors. Differences between the first and the last time points were tested by chi-square distribution and had values of *P*<0.05 (**B**), Comparison of overall transcript levels between the first 4 time points and the last 5 time points shows significant decrease in transcription levels of subtelomeric genes over time (*P = 0.005*; Mann-Whitney U test) (**C**), Analyzing the transcription data for underlying switch dynamics of each individual *var* gene over half a year after switching initiation demonstrates biased activation and silencing patterns. The *var* gene repertoire can be envisaged as a switching network whose nodes represent individual *var* genes and edges the probability of a switch between them [19]. The strong negative correlation between a variant's in - and out - degree persists even after removing a large proportion of the network, thus supporting the non-random pattern of *var* gene switching in our data.

**Figure 4:**
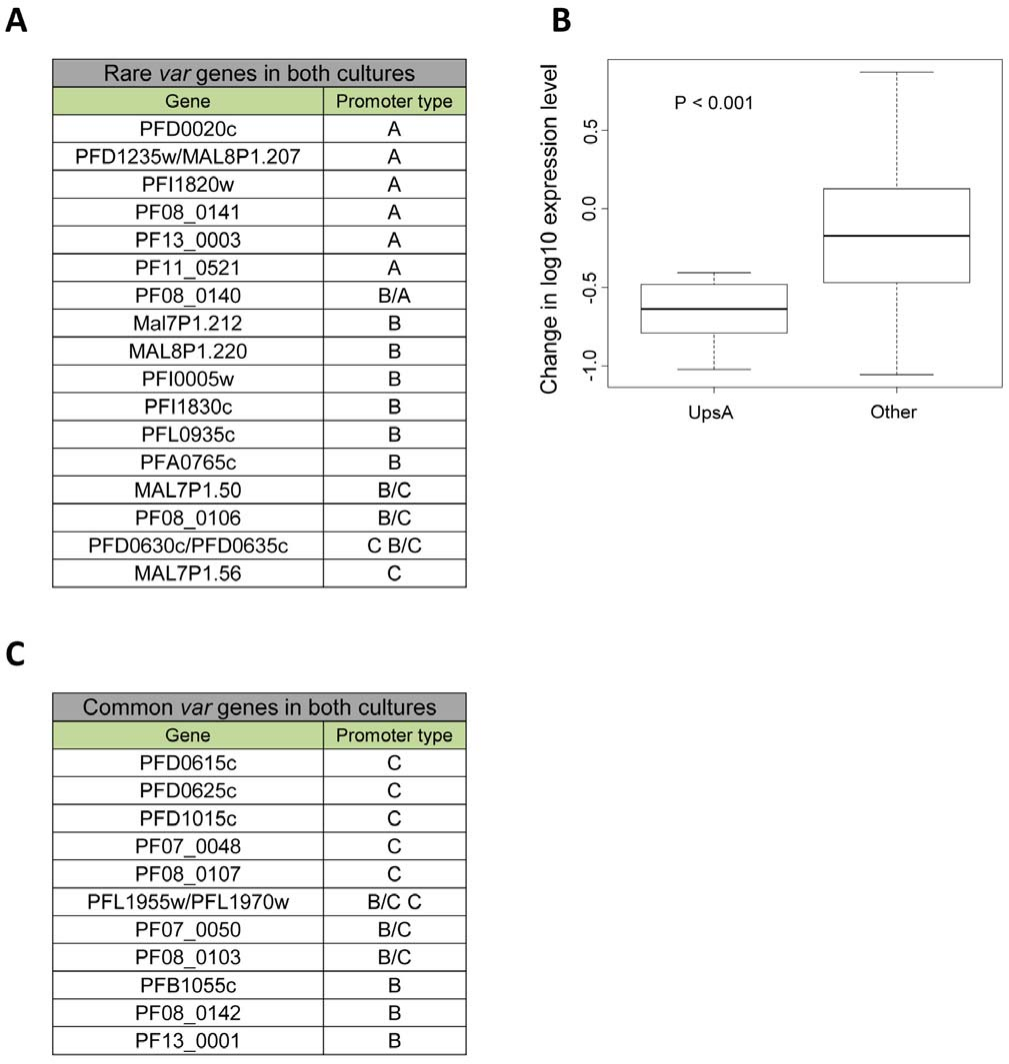
Highly and rarely activated *var* genes. *var* gene were sorted by their relative mRNA levels to the total *var* transcript of the entire family, at each time point for all three populations. The *var* genes that were common among both populations at least twice are displayed. (**A**), *var* genes that are rarely activated. Note that among the 19 *var* genes that are rarely activated, 14 are subtelomeric. In addition, *var* genes with upsA promoters are highly represented in this group. (**B**), UpsA *var* genes become less transcribed over time. Comparison of overall upsA transcript levels between the first 4 time points and the last 5 time points shows significant decrease in transcription levels of upsA *var* genes over time (*P = 0.007*; Mann-Whitney U test (**C**), *var* genes that have high probability to be activated. Among the 12 *var* genes that are commonly activated internal *vars* with upsC promoters are highly represented.

**Figure 5:**
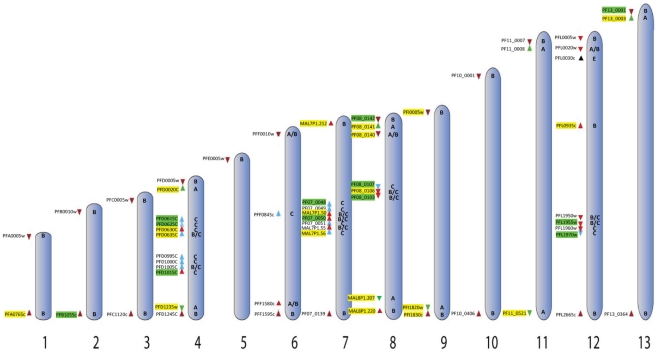
Schematics of the *var* gene family in the NF54 genome presenting the chromosomal location of *var* gene that are highly or rarely activated. Chromosomes containing *var* genes are numbered, and each arrowhead represents a single *var* gene (modified from: [46]). Accession numbers and promoter type are marked for each individual gene. *var* genes that have high probability to be activated are displayed in green and *var* genes that are rarely activated are in yellow.

The dynamics of switching for each of the *var* subsets showed a bias in the relative transcription over-time towards particular genes in each population due to hierarchical switch order (Fig. 3). These results indicate that the switching patterns of parasites with no previous memory are similar to switches of clonal populations with previous epigenetic memory of *var* expression [17], [18], [19]. However, contrary to what was previously concluded, our gene specific analyses of the transcription time courses imply that promoter type or chromosomal location *per se* could not explain these dynamics. Instead, intrinsic properties within each particular gene seem to underlie the observed dynamics. Over time, there was an increase in the ratio of the upsC type in the G-6 populations and an increase in the upsB/C in the E-1 population. At the same time we observed a significant decrease in the relative levels of transcripts from the upsA promoter type in both populations (Fig. 1 & 2 and Fig. S1 & S2). These results indicate that while some particular *var* genes have a high probability to be activated and dominate the culture over time, other *var* genes are rarely activated. In order to test whether the genes that are rarely activated share common characteristics, we sorted all *var* genes by their transcription levels at each time point after the first month of switching initiation in both populations. We scored the genes in which their cumulative transcripts represent the lowest fifth percentile of the total *var* transcripts in the population. This was done for both populations at each time point. We found that 19 of the genes that are rarely activated are common in both populations (Fig. 4A). In addition, it appears that *var* genes with upsA promoters are highly represented in this group (7/8 upsA *var* genes) (Fig. 4B). Moreover, we noted that some of the upsC *var* genes (MAL7P1.50, MAL7P1.56, PFD0630c/0635c and PF08_0106) that are found in gene's clusters in internal chromosomal loci are also rarely activated even though the adjacent genes in the cluster are amongst the most highly activated genes in the genome. Similarly, we scored the highly activated *var* genes as the ten most active genes in both clones at each time point from one month throughout the entire experiment. We found that 12 of the most active genes are common for both clones and that most of them are upsC (Fig. 4C). This suggests that a large proportion of the *var* repertoire has low probability to be expressed *in vitro*. In addition, while most upsA *var* genes are rarely activated, in other promoter types some genes are highly activated and some are rarely activated. This implies that switch rates are determined by gene specific associated mechanisms rather than a general promoter type or chromosomal locus associated mechanism. Therefore, we concluded that the switching properties of each *var* gene are independent of its chromosomal location or promoter type *per se* (Fig. 5).

### Partial var repression promotes switching but does not erase the epigenetic memory

**Figure 6:**
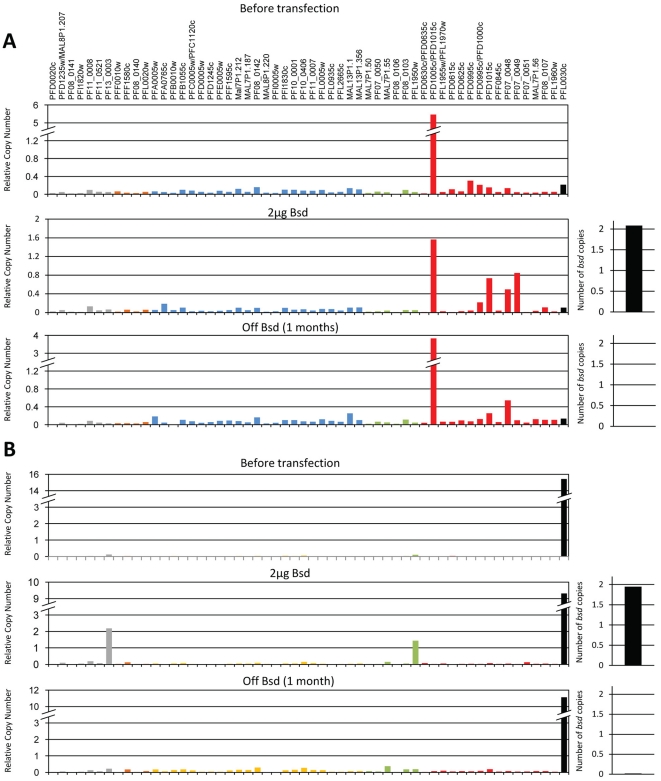
Low levels of promoter titration that reduce transcription promote switching but do not erase the epigenetic memory. Two clonal populations G-6 (**A**) and CSA (**B**) (upper panels) were transfected with the plasmid pVBH and selected on low dose of 2 µg/ml blasticidin (middle panels). These populations carried two copies of the competing episomes (right diagram). Under drug selection the transcription levels of the genes that were exclusively expressed prior to titration: PFD1005c and PFL0030c were reduced in both the G-6 and the E-1 respectively, while transcription of other *var* genes was detected. A month after the removal of drug pressure, both populations returned to their previous clonal expression patterns (lower panels). All values are presented as relative copy number to the housekeeping gene arginyl-tRNA synthetase (PFL0900c). *var* genes are colored by promoter type: Grey, upsA; Orange, upsA/B; Blue, upsB; Green, upsB/C; Red, upsC; Black, upsE.

Passing a certain threshold of transcriptional down-regulation of *var* genes by promoter titration has been shown to result in the loss of their epigenetic memory [22]. However, the effect of only a partial repression of the endogenous gene by low copy number of competing episomes is unknown. We reasoned that if the maintenance of epigenetic memory is associated with the act of transcription *per se* then it is possible that transcriptional interference by fine tuned promoter titration will trigger faster switching to other genes. We therefore analyzed the entire *var* transcription profile of two parasite lines with low switch rate: the G-6 and CSA-selected populations after transfection with pVBH and subsequent selection on low dose of only 2 µg/ml blasticidin. Under this low drug pressure these parasite populations carried only two copies of the competing pVBH episome (Fig. 6) and transcription levels of PFD1005c and PFL0030c were down-regulated by 2–3 fold; however, both remained the dominant *var* genes in the G-6 and the CSA-selected populations respectively.

In both clones the reduced transcription levels of the dominant *var* gene was associated with the appearance of detectable transcripts from other endogenous genes (Fig. 6). In both the G-6 and the CSA-selected populations that were not transfected with pVBH and kept growing in culture during the course of the experiment, no apparent switches were observed and their transcription profiles remained similar to the original clonal populations (Fig. S3). These results indicate that the observed switches to other *var* genes were specific to the populations in which transcription levels of the dominant *var* genes were reduced by titration. Moreover, the fact that under such low levels of promoter titration, PFD1005c and PFL0030c remained the dominant *var* genes suggests that the epigenetic memory that marks these genes as the active genes is still maintained. To test this hypothesis we simply removed drug pressure and let these populations remove the competing episomes. We found that contrary to the promoter titration with multi-copy pVBH that completely erased the epigenetic memory and resulted in heterogenous *var* transcription patterns, both the G-6 and the CSA-selected populations reverted back to clonal transcription patterns in which each of the populations was expressing exclusively either PFD1005c or PFL0030c respectively (Fig. 6). These results suggest that the epigenetic memory is weakened when transcription levels are repressed below the threshold that leads to complete silencing and that this partial repression can induce higher switch rates.

## Discussion

Antigenic variation has evolved in many pathogens as a successful mechanism to evade host immunity and to sustain long-term chronic infections [24]. This is especially important for vector-born pathogens such as *P. falciparum* in order to ensure the continuity of transmission in regions where mosquito abundance is seasonal. Antigenic variation created by *P. falciparum* is tightly regulated by continuous switches between expressions of different *var* genes. It has been proposed that different *var* genes turn on and off at different rates [18], [19], [25]. These studies were able to demonstrate that the history of expression in a clonal population expressing a particular *var* gene would affect the future switching pattern in the population and proposed a model of hierarchical switching order which would help the parasite to avoid fast exposure of its antigenic repertoire. However, since *var* genes are expressed exclusively during the intra-erythrocytic development cycle (IDC) and not in liver stages, the switching patterns that occur at the onset of malaria when the first merozoites burst out of hepatocytes and start replicating within iRBC begin from a null-*var* background rather than from parasites expressing any particular *var* gene. Here we used a controlled *in vitro* system to erase the epigenetic memory and create a null-*var* background in blood stages parasites. This allowed us to study the switching patterns from the onset of switching and over a period of six months. Our results showing a heterogeneous *var* expression at the onset of switching is similar to the parasite population recovered from a patient only 11 days post infection [20] and may indicate that merozoites leaving the liver with a null-*var* background employ similar epigenetic marks to intra-erythrocytic parasites.

We have previously proposed that in clonal populations, switches in expression of subtelomeric *var* genes are more frequent than the switches of *var* genes that are located in internal regions of the chromosome, which led to the conclusion that the intrinsic switch rate of a gene is correlated with its chromosomal position [18]. The results of our current study imply that many of the genes that are located in internal loci, mainly those with upsC promoters, have a high probability to be activated. However, some of the most highly activated genes in both clones are subtelomeric *var* genes. In addition, we found that genes with low activation probability are located mostly in subtelomeric regions, but a few are found in the internal gene clusters and have either upsC or upsB/C promoters. This clearly indicates that a simple model in which the chromosomal location or the type of promoter *per se* determines the fate of a *var* gene does not explain the differences in the probability of a particular *var* gene to get activated. Rather, this indicates for a gene specific associated mechanism that determines its switch rate. It is still possible that the odds of each particular gene to get activated depends on its position in the overall switching network as previously suggested [19]. These authors also demonstrated that the hierarchies of switching often show patterns that favor switching to genes that are located at different chromosomes. Such switching hierarchies could also explain why the *var* subtypes of genes that were predominantly transcribed in cultures after half a year in both the E-1 and G-6 clones were similar to those of the original clonal populations before erasing their memory. We show that some of the genes that are highly activated and those that are rarely transcribed are located adjacent to each other at the same chromosomal locus. Remodeling the chromatin structure was implicated in *var* gene regulation; however, having the two extremes, highly and rarely activated genes, at the same locus suggests that switching does not necessarily occur through the spreading of euchromatin from the active gene to the adjacent gene. In addition, our results support a model by which each *var* gene is an autonomous unit that contains all DNA elements that are required to regulate antigenic switching and mutually exclusive expression. Indeed, recombination in the *var2csa* regulatory intron had altered its switch rate [26].

In order to silence *var* genes and to erase the epigenetic memory it has been proposed that the level of transcriptional repression needs to be below a certain threshold. This implied that active transcription is required for the maintenance of the epigenetic memory that regulates *var* gene expression [22] possibly through the involvement of PolII [27]. However, the exact mechanism by which the act of transcription is involved in maintaining the epigenetic memory is unknown. It is possible that by reducing the level of transcription of a particular gene as demonstrated here, the maintenance of the epigenetic marks required for epigenetic memory were also weakened which in turn increased the probability of expression to switch to another gene. This could explain why in the populations subjected to fine tuned transcriptional repression additional *var* transcripts were detected, since there is no evidence that the mechanisms of mutually exclusive expression were interrupted in those parasites. How these parasite populations revert back to the original dominant gene is yet to be discovered, however, similar phenomenon was recently reported in parasite populations switching *in vitro* without prior manipulation of the epigenetic memory [25]. Further investigation is needed to explore the possibility that some *in vivo* situations may perturb *var* transcription and alter antigenic switching.

Various attempts have been made to link *var* gene expression with the clinical manifestations of malaria and to investigate the role of specific PfEMP1 variants with pathogenesis. Profiling *var* expression in field isolates taken from Kenyan children showed an association between the abundance of specific *var* transcripts and the “rosetting” properties of the iRBC [28]. Cerebral malaria was also associated with specific PfEMP1 expression. Parasites that were isolated from organs of children dying from malaria showed clear dominance of a small number of certain *var* transcripts in parasites that were sequestered in the brain. This suggests that PfEMP1 proteins encoded by only a small number of *var* genes are responsible for sequestration in the brain microvasculature [29], possibly through specific binding to ICAM1 [30], [31]. Furthermore, using *in vitro* selection on sera taken from children with severe malaria it was shown that parasites that cause severe childhood malaria preferentially express PfEMP1 variants that are encoded by upsA *var* genes [32]. UpsA *var* genes were found to be predominantly transcribed in children with cerebral malaria [33] and a particular upsA *var* gene (PF13_0003) was recently linked to the “rosetting” phenotype [34]. We found that upsA *var* genes are activated at the onset of switching similar to other *var* subtypes but quickly switch away in growing cultures. Moreover, transcripts from these genes did not appear in any of the clones even after half a year in culture suggesting a very low on rate outside the human host. However, we did not obtain any upsA expressing clone and therefore could not investigate the effect of promoter titration on such parasite line. UpsA *var* genes have been shown to switch away during adaptation of wild-type parasites to culture [35] and seem to be rarely activated in clones of the IT line [36]. Recent computational analysis demonstrated that upsA *var* genes maintain a high level of conservation of specific PfEMP1 blocks across several genetic lines [37]. The low probability of upsA *var* genes to be activated and their association with severe malaria might offer a possible explanation for this relatively high degree of conservation. In chronic infections upsA *vars* are rarely activated and therefore are not exposed to immune pressure. However, when upsA *var* genes are activated in non-immune individuals they would lead to severe malaria that would either have fatal consequences or immunity acquisition. This explanation is supported by a clinical study that showed that PfEMP1 variants encoded by upsA *var* genes with cytoadhesive properties that confer a growth advantage dominate infections in naïve children in the absence of a protective immune response [38], while the cytoadhesive properties of these variants lead to severe malaria. The authors suggested that rapid immunity acquisition to this relatively conserved group may explain why children are less susceptible to severe malaria after few infections [39]. While it is possible that at the onset of switching activation of upsA *var* genes could lead to severe malaria in the absence of immunity by cytoadhesive selection. Our results may suggest that evolutionary evolvement of the high “off” rates of these *var* genes enables the parasites to evade immune attack in pre-immune patient by pre-programmed rapid switching to less conserved PfEMP1. Understanding antigenic gene expression at the onset of malaria as well as host-parasite interactions that influence parasites' establishment and pathogenicity at the early phase of infection will hopefully lead to the development of new intervention strategies.

## Materials and Methods

### Parasite Culture

All parasites used were derivatives of the NF54 parasite line and were cultivated at 5% hematocrit in RPMI 1640 medium, 0.5% Albumax II (Invitrogen), 0.25% sodium bicarbonate, and 0.1 mg/ml gentamicin. Parasites were incubated at 37°C in an atmosphere of 5% oxygen, 5% carbon dioxide and 90% nitrogen. Clonal populations were obtained by limiting dilutions as previously described [18]. Each population expressed a different *var* gene: the G6 clone expressed PFD1005c; the E1, predominantly expressed PFL0005w and CSA exclusively express PFL0030c.

Parasite cultures were synchronized using percoll/sorbitol gradient centrifugation as previously described [40], [41]. Briefly, infected RBCs were layered on a step gradient of 40%/70% percoll containing 6% sorbitol. The gradients were then centrifuged at 12,000 g for 20 minutes at room temperature. Highly synchronized, late stage parasites were recovered from the 40%/70% interphase, washed twice with complete culture media and placed back in culture.

### Plasmid Construction and Parasite Transfection

The plasmids pVBH, is described [22]. Parasites were transfected as described [42]. Briefly, 0.2 cm electroporation cuvettes were loaded with 0.175 ml of erythrocytes and 100 µg of plasmid DNA in incomplete cytomix solution. Stable transfectants were initially selected on 2 µg/ml blasticidin (Invitrogen, USA). In order to obtain parasites carrying large plasmid copy numbers, these cultures were then subjected to 10–15 µg/ml blasticidin for two weeks or until normal growth rates were observed. At this stage parasite were grown to 5% parasitemia in 20 ml culture for RNA extraction.

### Genomic DNA extraction, RNA extraction and cDNA synthesis

Genomic DNA was extracted as described [22]. Briefly, iRBCs were pelleted and lysed with Saponin. Parasites from 20 ml cultures were pelleted, washed with PBS, and taken up in 200 µl TSE buffer (100 mM NaCl, 50 mM EDTA, 20 mM Tris, pH 8), 40 µl of 10% SDS and 20 µl 6 M NaClO_4_. This suspension was rocked for twelve hours and genomic DNA was extracted with phenol/chloroform. The resulting DNA was taken up in 100 µl dH_2_O.

RNA extraction and cDNA synthesis was performed as described [16]. Briefly, RNA was extracted from synchronized parasite cultures at 18 hours post invasion, corresponding with the peak RNA expression levels for determination of *var* transcript levels. RNA was extracted with the TRIZOL LS Reagent® as described [43] and purified on PureLink column (Invitrogen) according to manufacturer's protocol. Isolated RNA was then treated with Deoxyribonuclease I® (Invitrogen) to degrade contaminating gDNA. cDNA synthesis was performed from 800 ng total RNA with Superscript II Rnase H reverse transcriptase ® (Invitrogen) with random primers ® (Invitrogen) as described by the manufacturer.

### Real-time RT-qPCR

For RT-qPCR reactions to detect transcription from all *var* genes present in the 3D7 genome, we used the primer sets published by Salanti et. al. [44] with few modifications published in [45]. Transcript copy numbers were determined using the formula 2^−ΔΔCT^ as described in the Applied Biosystems User Bulletin 2 using NF54 gDNA as the calibrator. Specifically, relative copy number was calculated as 2 exponential negative ((Ct target gene in cDNA – Ct reference gene in cDNA)-(Ct target gene in gDNA-Ct target gene in gDNA)). Copy numbers of episomal promoters were calculated using RT-qPCR of gDNA and comparing the ΔCT of *bsd* to that of the single copy housekeeping gene arginyl-tRNA synthetase (PFL0900c). This gene as well as P60-seryl-tRNA synthetase (PF07_0073), P61-fructose bisphosphate aldolase (PF14_0425), P100-actin (PFL2215), and glutaminyl-tRNA synthetase (PF13_0170) were used as control genes in all RT-qPCR assays as described [45]. The reference gene used for result presentations in all of the presented graphs presented in the manuscript is arginyl-tRNA synthetase. All RT-qPCR assays were performed at least in duplicate for each template with no apparent differences, and each experiment was completed at least twice in its entirety, again with no significant differences.

### Var gene transcription analysis

We used the same computational method previously employed by Recker et al., [19] to analyze the long-term transcription profiles. In brief, *var* gene switching can be described simply by the rate an active gene becomes silent, *r_i_*, and the probability of a switch from gene *i* to gene *j*, *b_ij_*.The method then determines the switch rates and switch biases that best fit the experimental data and which can then be expressed as an overall, directed switching network where the nodes represent the individual genes and the edges the net transition between them, i.e. the difference r_i_*b_ij_ – r_j_*b_ji_. Because of the deterministic nature of this method the resulting network is fully connected and will naturally exhibit a negative correlation between a node's out-degree, i.e. the number of genes it switches to, and its in-degree, i.e. the number of genes that switch towards this gene. To investigate the robustness of this correlation we therefore progressively removed edges with the weakest transition rates and compared the resulting correlation to a randomly connected network with a comparable number of connections removed.

## Supporting Information

Figure S1 Switching dynamics of parasite population expressing a subtelomeric *var* gene (E-1) in the following generations after erasing its epigenetic memory. (**A**), Steady state mRNA levels of each individual *var* gene are presented as relative copy number. Each *var* gene is colored by its promoter type: Grey, upsA; Orange, upsA/B; Blue, upsB; Green, upsB/C; Red, upsC; Black, upsE. Transcription patterns are presented at different time points after drug removal and switching initiation: two months (upper panel), four months (second panel), and six months (lower panel). Transcription levels were measured by RT-qPCR. All values are presented as relative copy number the to the housekeeping gene arginyl-tRNA synthetase (PFL0900c). (**B**), The relative proportion of transcripts of each *var* gene subsets, A, A/B, B, (telomeric) and B/C, C, (central) from the total *var* transcripts is displayed for each time points as a pie chart on the right. The relative proportion of transcript was normalized to the number of genes belonging to each *var* subset.
(TIF)

Figure S2 Switching dynamics of parasite population expressing an internal *var* gene (G-6) in the following generations after erasing its epigenetic memory. (**A**), Steady state mRNA levels of each individual *var* gene are presented as relative copy number. Each *var* gene is colored by its promoter type: Grey, upsA; Orange, upsA/B; Blue, upsB; Green, upsB/C; Red, upsC; Black, upsE. Transcription patterns are presented at different time points after drug removal and switching initiation: two months (upper panel), four months (second panel), and six months (lower panel). Transcription levels were measured by RT-qPCR. All values are presented as relative copy number to the housekeeping gene arginyl-tRNA synthetase (PFL0900c). (**B**), The relative proportion of transcripts of each *var* gene subsets, A, A/B, B, (telomeric) and B/C, C, (central) from the total *var* transcripts is displayed for each time points as a pie chart on the right. The relative proportion of transcript was normalized to the number of genes belonging to each *var* subset.
(TIF)

Figure S3 *Var* transcription patterns of the un-transfected clonal populations after one month of *in vitro* switching. Both the untransfected G-6 (**A**) and the CSA-selected (**B**) populations used for the fine tune down regulation experiment on 2 µg/ml blasticidin presented in figure 6 were kept in culture during the course of the experiment. Steady state mRNA levels measured by RT-qPCR on cDNA of each individual *var* gene are presented as relative copy number. Each *var* gene is colored by its promoter type: Grey, upsA; Orange, upsA/B; Blue, upsB; Green, upsB/C; Red, upsC; Black, upsE. All values are presented as relative copy number to the housekeeping genes arginyl-tRNA synthetase (PFL0900c).
(TIF)
