# Supplementary figures and images for: Erasing the Epigenetic Memory and Beginning to Switch—The Onset of Antigenic Switching of var Genes in Plasmodium falciparum
Source: PLoS One. 2012 Mar 26;7(3):e34168. doi: 10.1371/journal.pone.0034168 (PMC3312910; doi:10.1371/journal.pone.0034168)

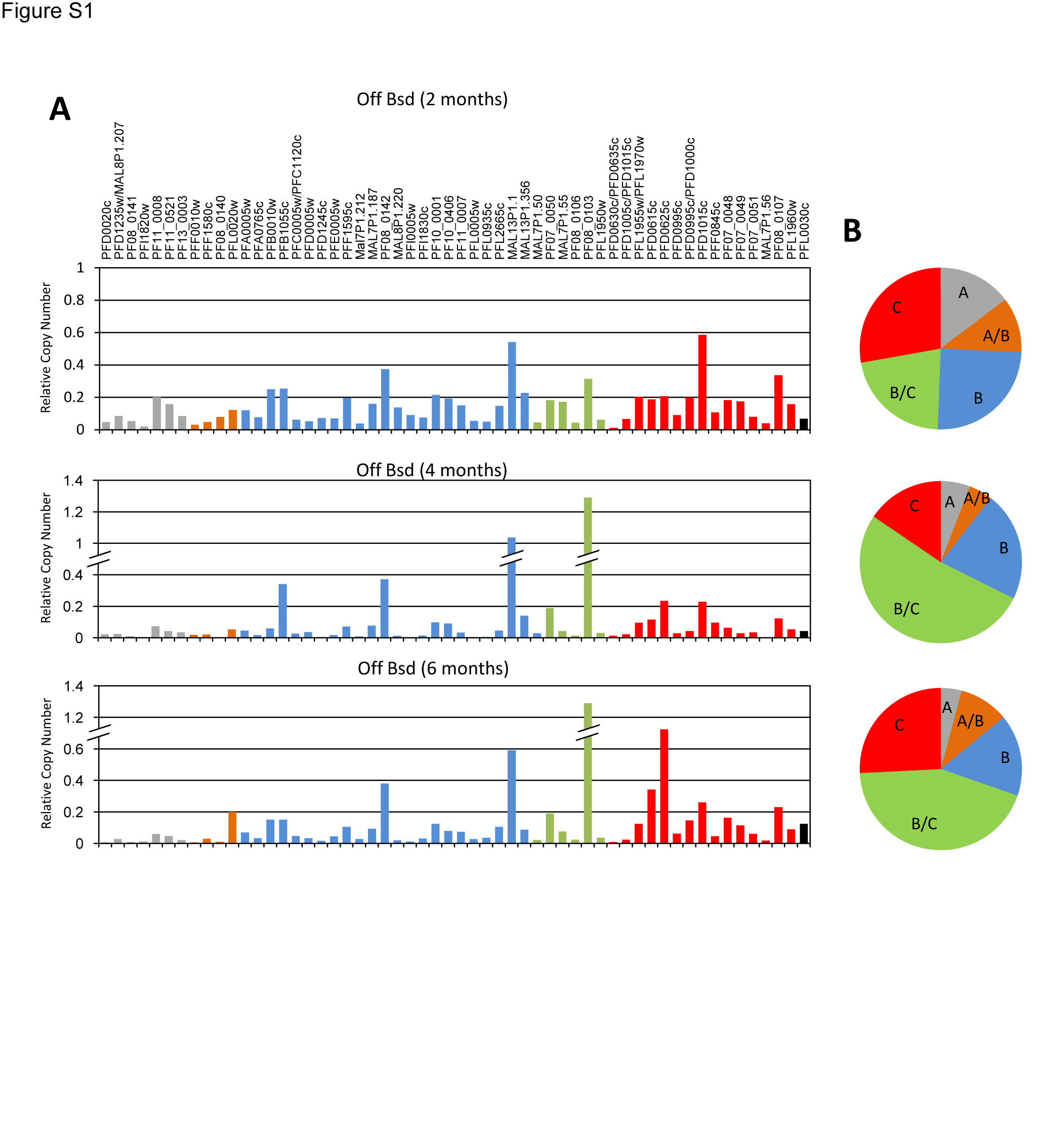

Supplement: Figure S1 — Switching dynamics of parasite population expressing a subtelomeric var gene (E-1) in the following generations after erasing its epigenetic memory. (A), Steady state mRNA levels of each individual var gene are presented as relative copy number. Each var gene is colored by its promoter type: Grey, upsA; Orange, upsA/B; Blue, upsB; Green, upsB/C; Red, upsC; Black, upsE. Transcription patterns are presented at different time points after drug removal and switching initiation: two months (upper panel), four months (second panel), and six months (lower panel). Transcription levels were measured by RT-qPCR. All values are presented as relative copy number the to the housekeeping gene arginyl-tRNA synthetase (PFL0900c). (B), The relative proportion of transcripts of each var gene subsets, A, A/B, B, (telomeric) and B/C, C, (central) from the total var transcripts is displayed for each time points as a pie chart on the right. The relative proportion of transcript was normalized to the number of genes belonging to each var subset. (TIF) [file pone.0034168.s001.tif]

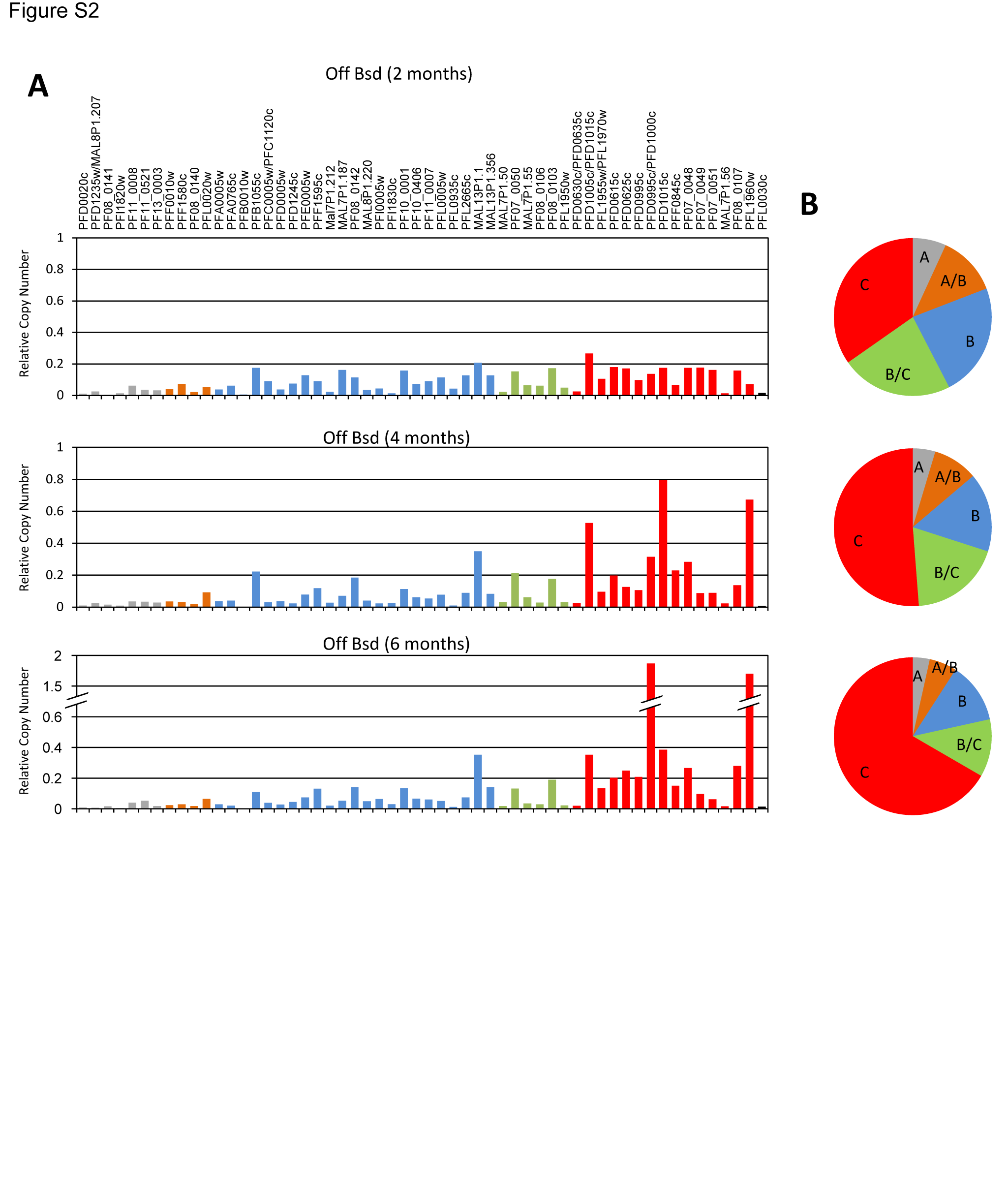

Supplement: Figure S2 — Switching dynamics of parasite population expressing an internal var gene (G-6) in the following generations after erasing its epigenetic memory. (A), Steady state mRNA levels of each individual var gene are presented as relative copy number. Each var gene is colored by its promoter type: Grey, upsA; Orange, upsA/B; Blue, upsB; Green, upsB/C; Red, upsC; Black, upsE. Transcription patterns are presented at different time points after drug removal and switching initiation: two months (upper panel), four months (second panel), and six months (lower panel). Transcription levels were measured by RT-qPCR. All values are presented as relative copy number to the housekeeping gene arginyl-tRNA synthetase (PFL0900c). (B), The relative proportion of transcripts of each var gene subsets, A, A/B, B, (telomeric) and B/C, C, (central) from the total var transcripts is displayed for each time points as a pie chart on the right. The relative proportion of transcript was normalized to the number of genes belonging to each var subset. (TIF) [file pone.0034168.s002.tif]

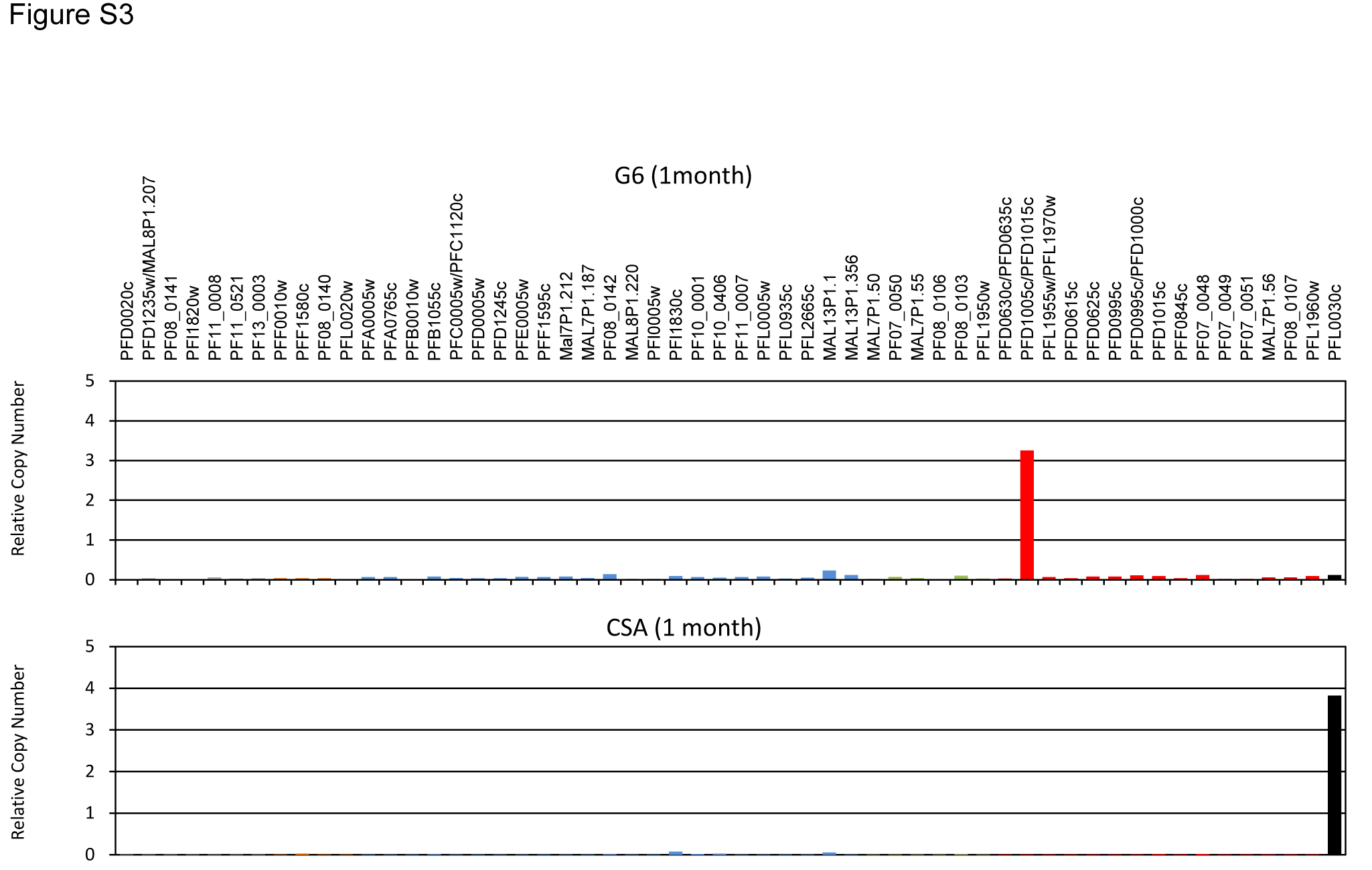

Supplement: Figure S3 — Var transcription patterns of the un-transfected clonal populations after one month of in vitro switching. Both the untransfected G-6 (A) and the CSA-selected (B) populations used for the fine tune down regulation experiment on 2 µg/ml blasticidin presented in figure 6 were kept in culture during the course of the experiment. Steady state mRNA levels measured by RT-qPCR on cDNA of each individual var gene are presented as relative copy number. Each var gene is colored by its promoter type: Grey, upsA; Orange, upsA/B; Blue, upsB; Green, upsB/C; Red, upsC; Black, upsE. All values are presented as relative copy number to the housekeeping genes arginyl-tRNA synthetase (PFL0900c). (TIF) [file pone.0034168.s003.tif]
